# P-649. Impact of rhinovirus in patients with different clinical presentation during 2023-2024 in Sao Paulo, Brazil

**DOI:** 10.1093/ofid/ofaf695.862

**Published:** 2026-01-11

**Authors:** Klinger S Faico-Filho, Ana Helena Perosa, Nancy Bellei

**Affiliations:** Universidade Federal de São Paulo, São Paulo, Sao Paulo, Brazil; Universidade Federal de São Paulo, São Paulo, Sao Paulo, Brazil; Universidade Federal de São Paulo, São Paulo, Sao Paulo, Brazil

## Abstract

**Background:**

Rhinovirus is a major cause of respiratory infections, commonly associated with symptoms such as the common cold.

This study aims to assess the occurrence of rhinovirus in patients with varying clinical presentations, including hospitalized adults and children, outpatients and asymptomatic individuals.

**Figure:**
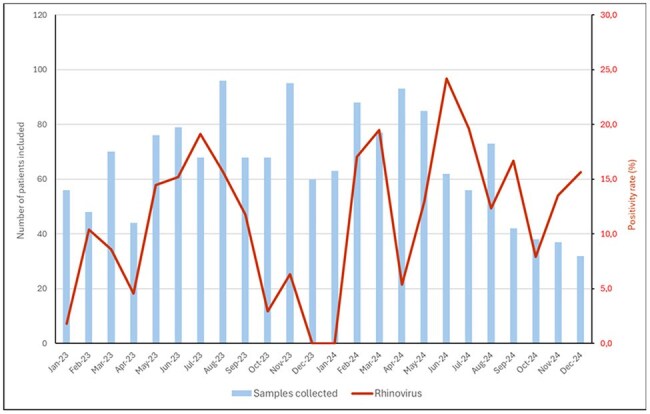
Monthly number of patients tested and rhinovirus positivity rate from January 2023 to December 2024.

Rhinovirus detection according to age groups and clinical presentation in adults participants.

**Figure:**
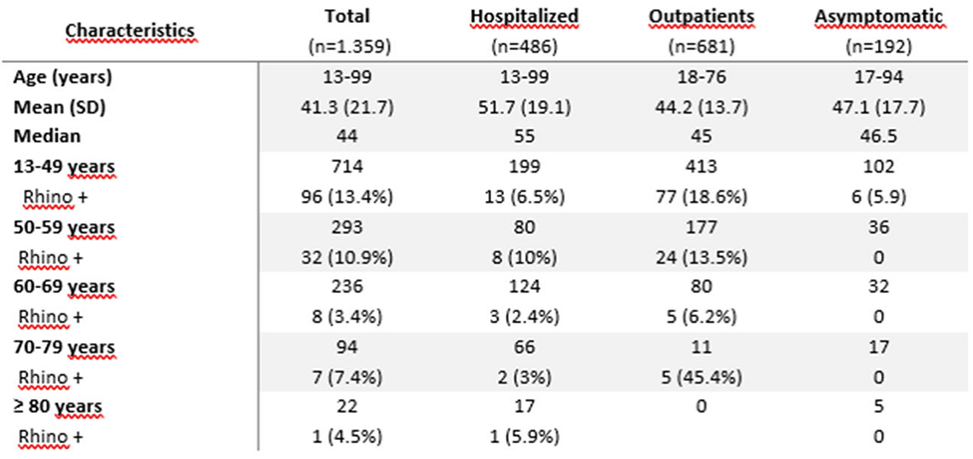

**Methods:**

A cross-sectional study was conducted with nasopharyngeal swabs collected between January 2023 and December 2024. A total of 1.574 participants were included and stratified into four groups: 486 hospitalized adults ( >12 years old) with lower respiratory tract infection (LRTI), 215 hospitalized children with LRTI, 681 health-care workers with acute respiratory infection attended at ambulatory and 192 asymptomatic individuals undergoing pre-surgical screening. Samples were tested for rhinovirus using RT-qPCR.

Characteristics and rhinovirus detection in hospitalized children.

**Figure:**
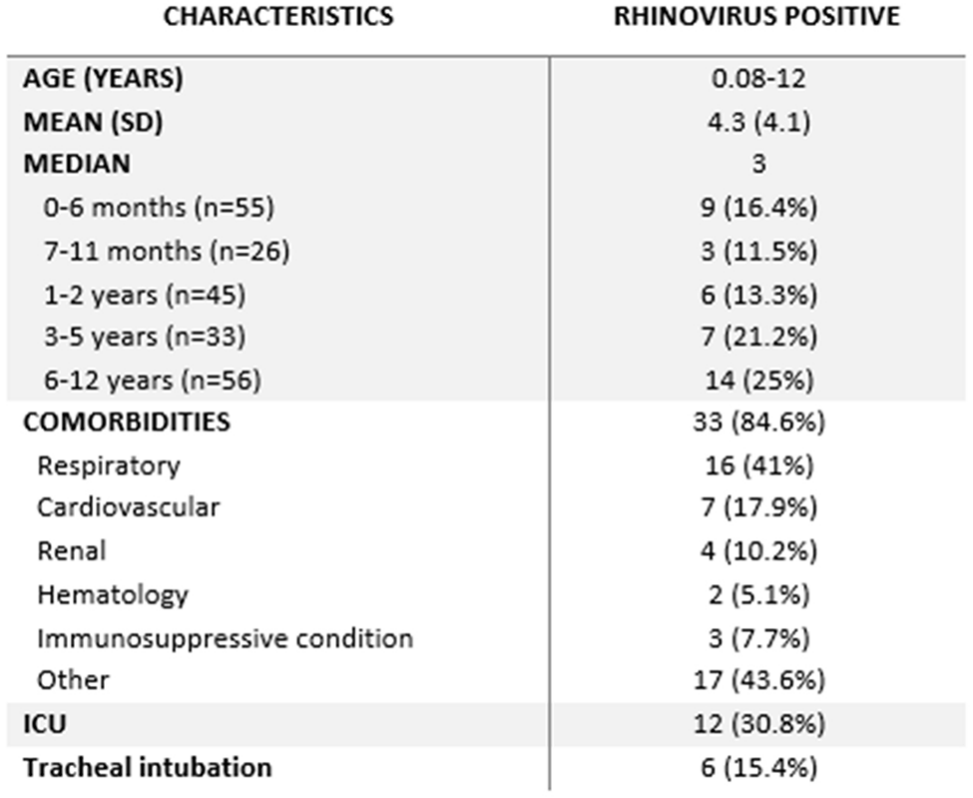

**Results:**

The prevalence of rhinovirus varied significantly across groups: 5.6% (27/486) in hospitalized adults, 18.1% (39/215) in hospitalized children, 16.3% (111/681) in ambulatory adults and 3.1% (6/192) in asymptomatic patients. In the 2 years analyzed, no seasonal pattern of circulation was observed (figure 1). Among hospitalized children with rhinovirus, 84.6% (33/39) had at least one comorbidity; 41% (16/39) respiratory disease, 17.9% (7/39) cardiovascular, 10.2% (4/39) renal, 5.1% (2/39) hematological, 7.7% (3/39) immunosuppressive condition and 43.6% (17/39) other comorbidities. Twelve children (30.8%) were admitted to the ICU and 6 (15.4%) needed tracheal intubation.

**Conclusion:**

Rhinovirus circulated year-round and was more prevalent in ambulatory adults and hospitalized children, especially those with comorbidities. Its detection in severe pediatric cases highlights its clinical impact beyond mild illness.

**Disclosures:**

All Authors: No reported disclosures

